# Supplementary material for: Lipidomic Profile Reconstruction of Therapeutic Membrane Targets Using Physics‐Based Optimization with Limited Activity Data
Source: Adv Sci (Weinh). 2026 Sep 3;13(62):e76899. doi: 10.1002/advs.76899 (PMC13542398; doi:10.1002/advs.76899)
Supplement: Supplementary file 2 [file ADVS-13-e76899-s001.zip › SI/9a7b7c11d6e0f2g10h2i2j3k14l12n14S0Z1C_flat.pdf]

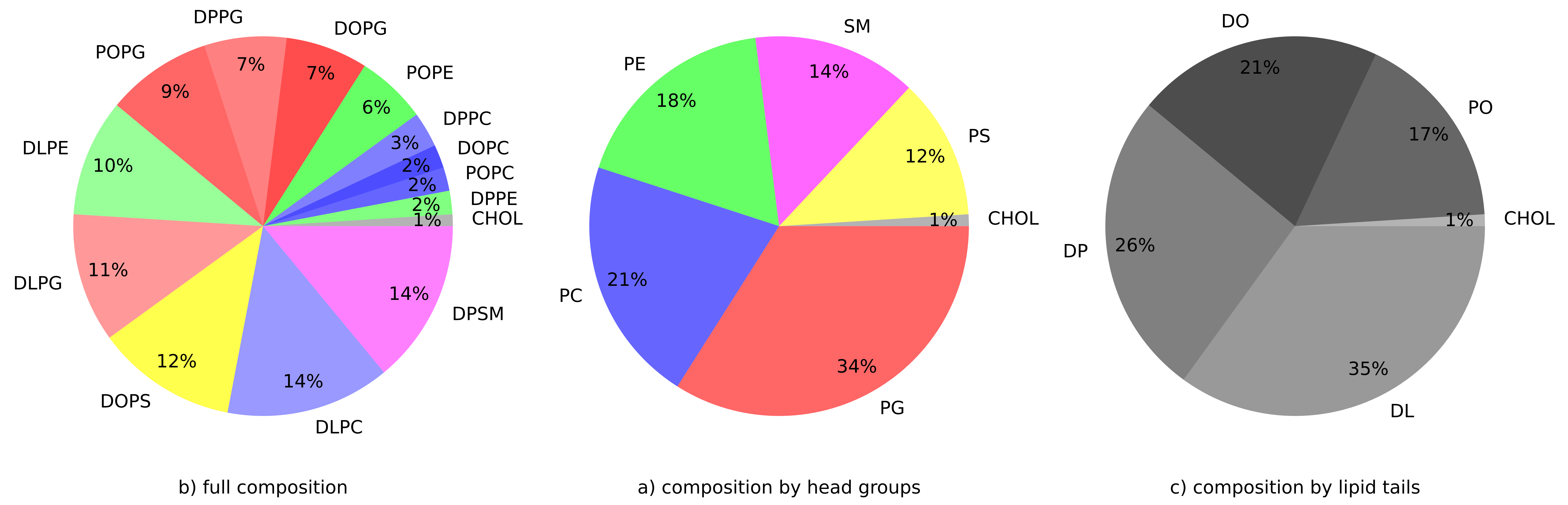
DPPG
DOPG
POPG
POPE
DPPC
DOPC
POPC
DPPE
CHOL
DLPE
DLPG
DOPS
DLPC
DPSM
7%
7%
9%
6%
3%
2%
2%
2%
1%
10%
11%
12%
14%
14%
b) full composition
SM
PE
PS
CHOL
PC
PG
14%
18%
12%
1%
21%
34%
a) composition by head groups
DO
PO
CHOL
DP
DL
21%
17%
1%
26%
35%
c) composition by lipid tails
