# Supplementary material for: Lipidomic Profile Reconstruction of Therapeutic Membrane Targets Using Physics‐Based Optimization with Limited Activity Data
Source: Adv Sci (Weinh). 2026 Sep 3;13(62):e76899. doi: 10.1002/advs.76899 (PMC13542398; doi:10.1002/advs.76899)
Supplement: Supplementary file 2 [file ADVS-13-e76899-s001.zip › SI/9a7b8c10d4e0f2g9h2i2j5k14l13n14S0Z1C_flat.pdf]

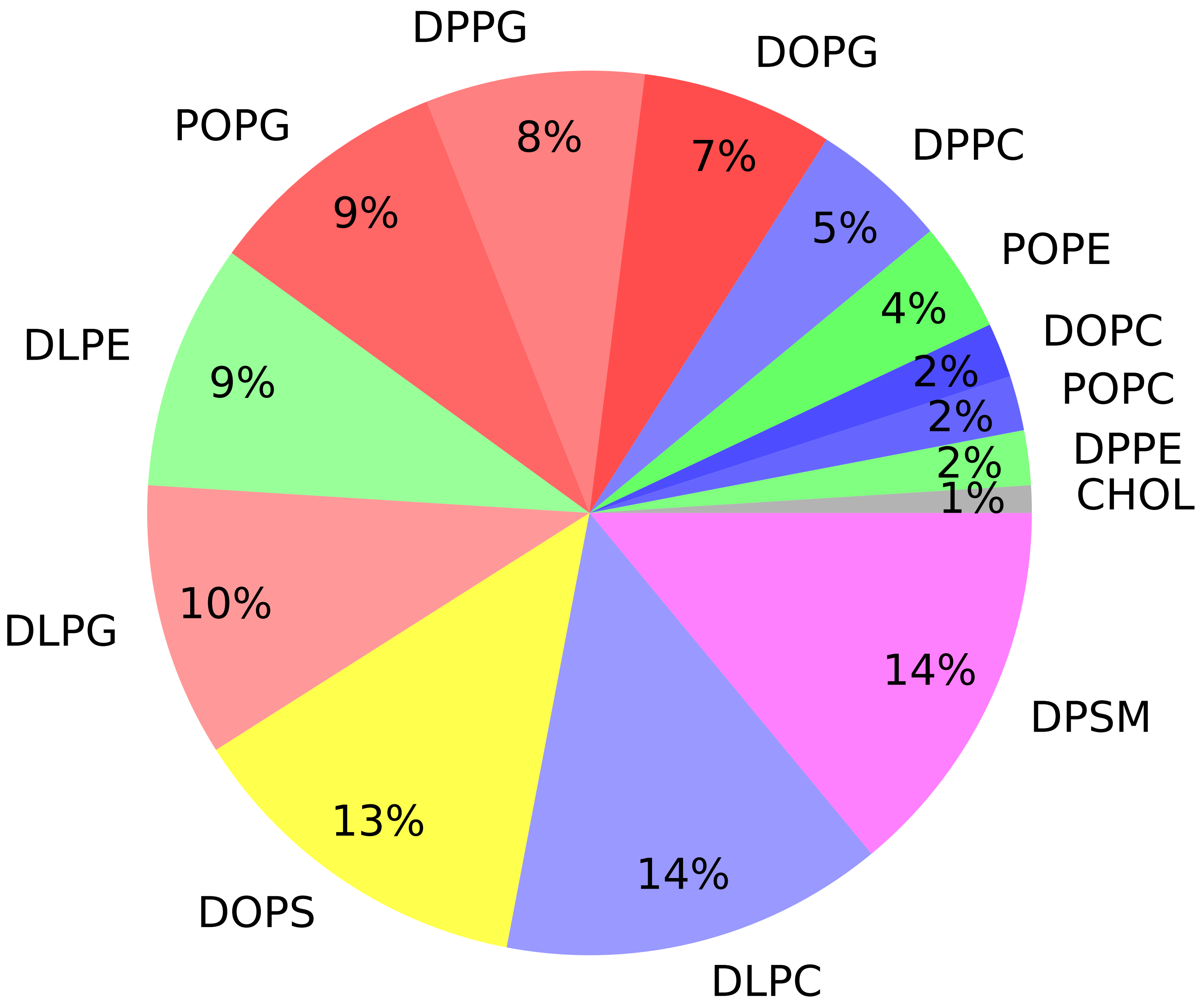

i) full composition

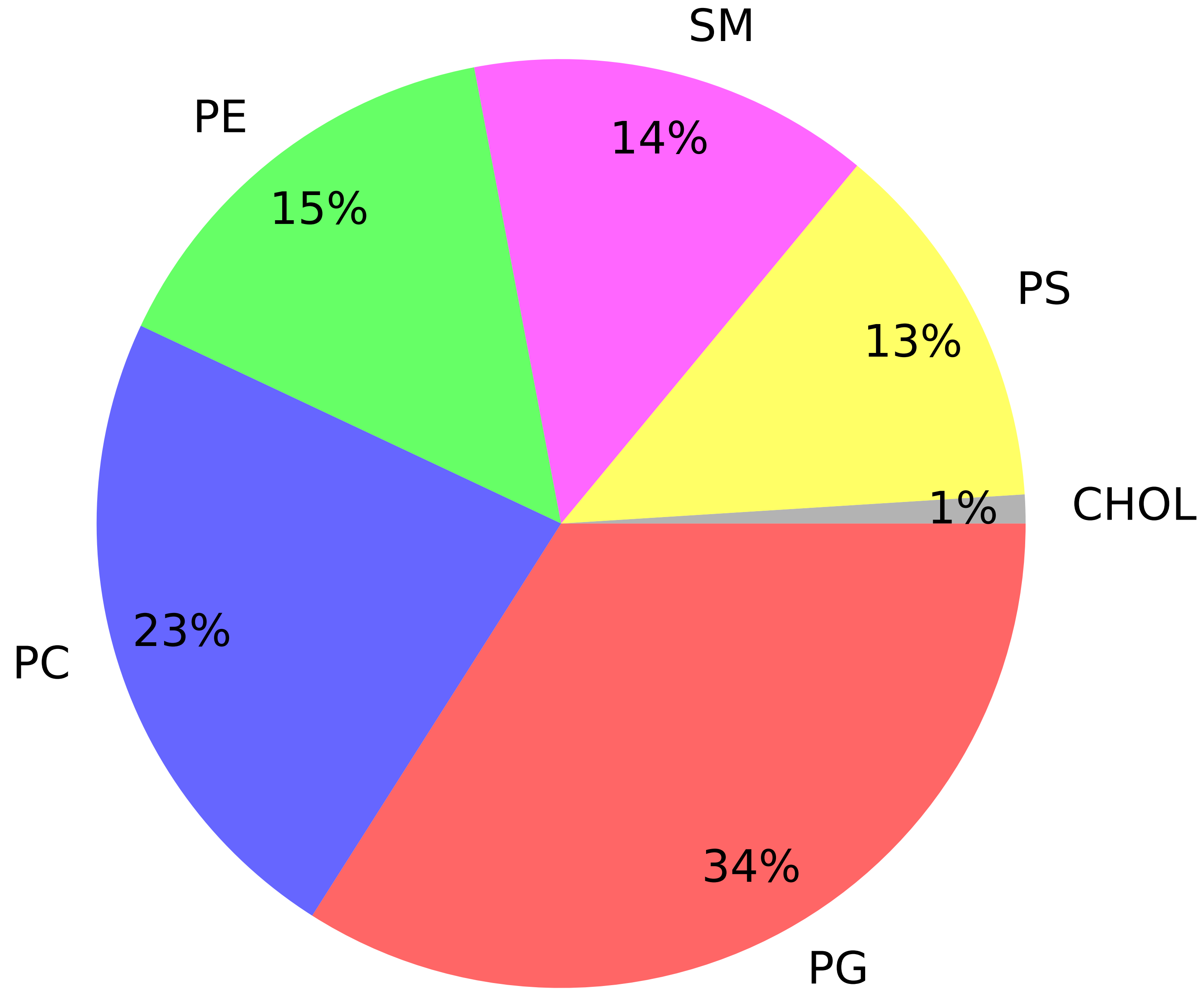

ii) composition by head groups

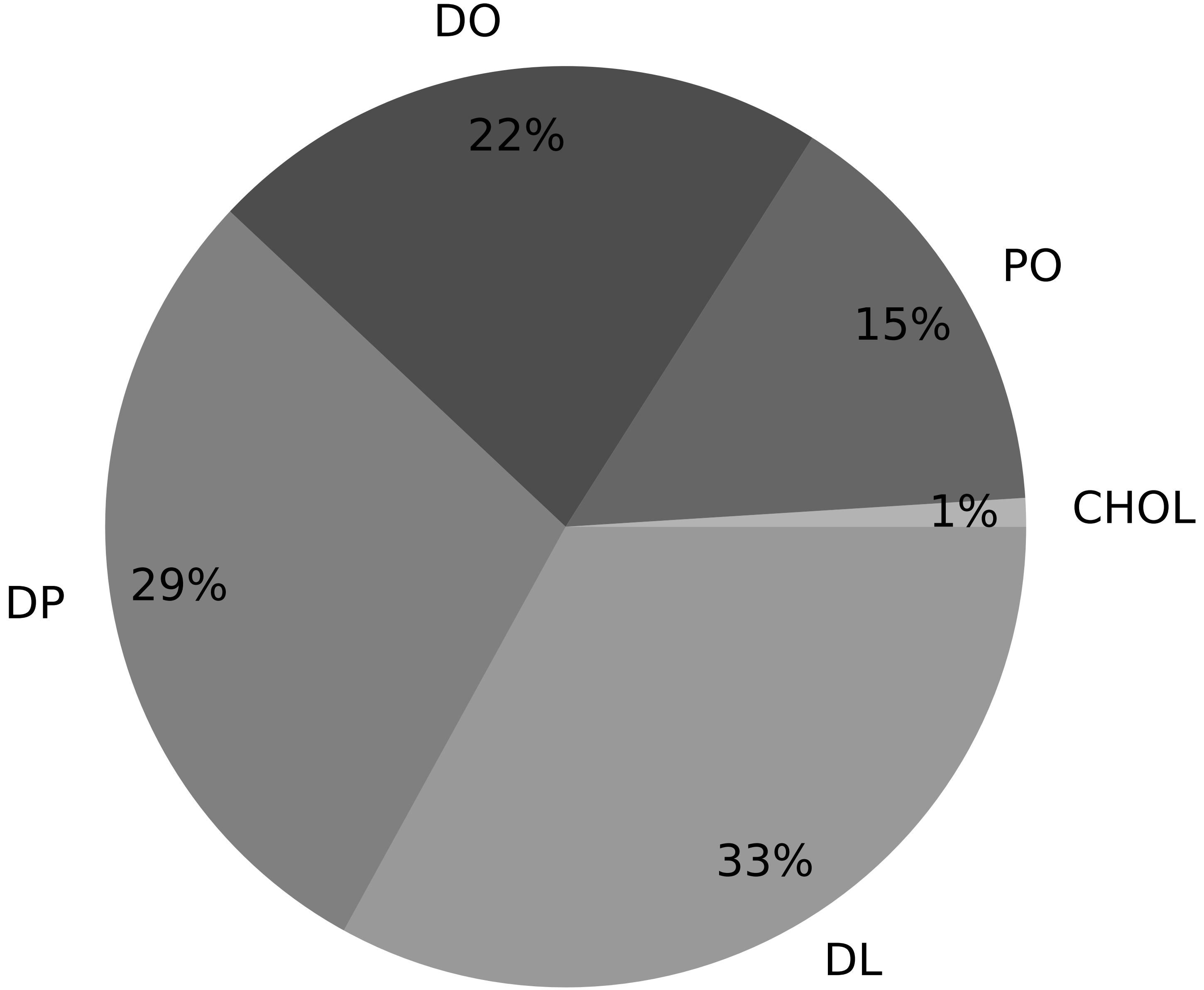

iii) composition by lipid tails
