# Supplementary material for: Lipidomic Profile Reconstruction of Therapeutic Membrane Targets Using Physics‐Based Optimization with Limited Activity Data
Source: Adv Sci (Weinh). 2026 Sep 3;13(62):e76899. doi: 10.1002/advs.76899 (PMC13542398; doi:10.1002/advs.76899)
Supplement: Supplementary file 2 [file ADVS-13-e76899-s001.zip › SI/plot.pdf]

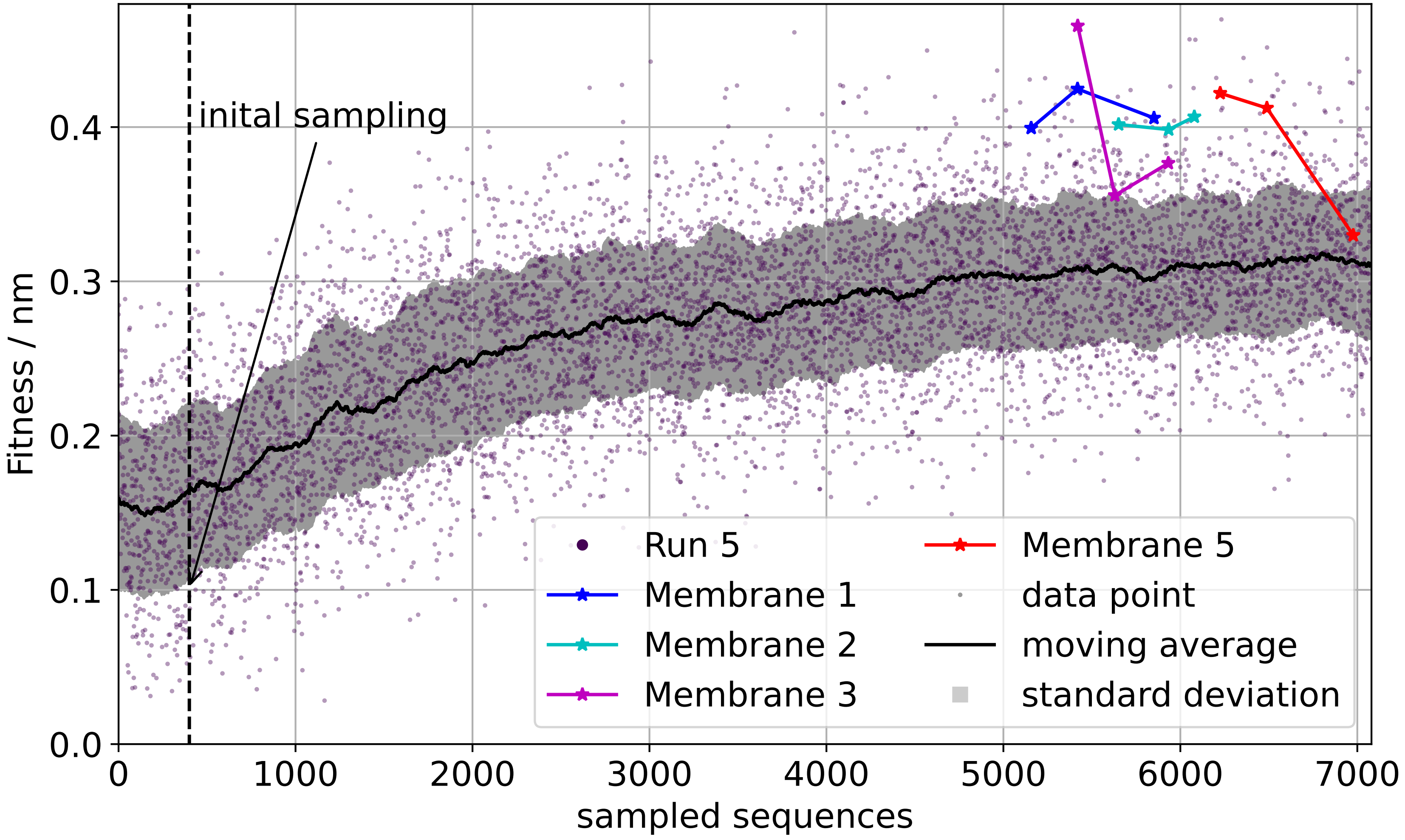

inital sampling
Fitness / nm
0.0
0.1
0.2
0.3
0.4
0
1000
2000
3000
4000
5000
6000
7000
sampled sequences
Run 5
Membrane 1
Membrane 2
Membrane 3
Membrane 5
data point
moving average
standard deviation
