# Supplementary material for: Lipidomic Profile Reconstruction of Therapeutic Membrane Targets Using Physics‐Based Optimization with Limited Activity Data
Source: Adv Sci (Weinh). 2026 Sep 3;13(62):e76899. doi: 10.1002/advs.76899 (PMC13542398; doi:10.1002/advs.76899)
Supplement: Supplementary file 2 [file ADVS-13-e76899-s001.zip › SI/pspg.pdf]

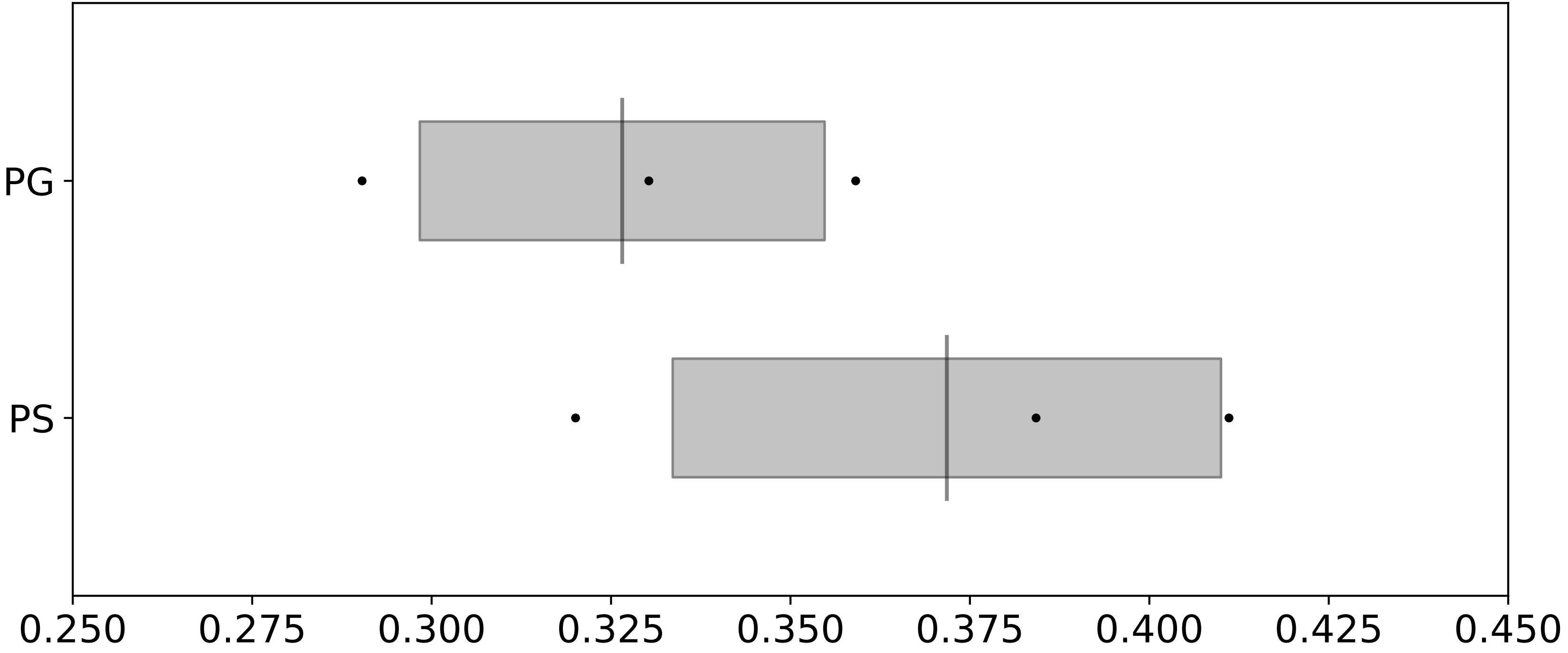

PG
PS
0.250
0.275
0.300
0.325
0.350
0.375
0.400
0.425
0.450
