# Supplementary material for: Lipidomic Profile Reconstruction of Therapeutic Membrane Targets Using Physics‐Based Optimization with Limited Activity Data
Source: Adv Sci (Weinh). 2026 Sep 3;13(62):e76899. doi: 10.1002/advs.76899 (PMC13542398; doi:10.1002/advs.76899)
Supplement: Supplementary file 2 [file ADVS-13-e76899-s001.zip › SI/quadratic.pdf]

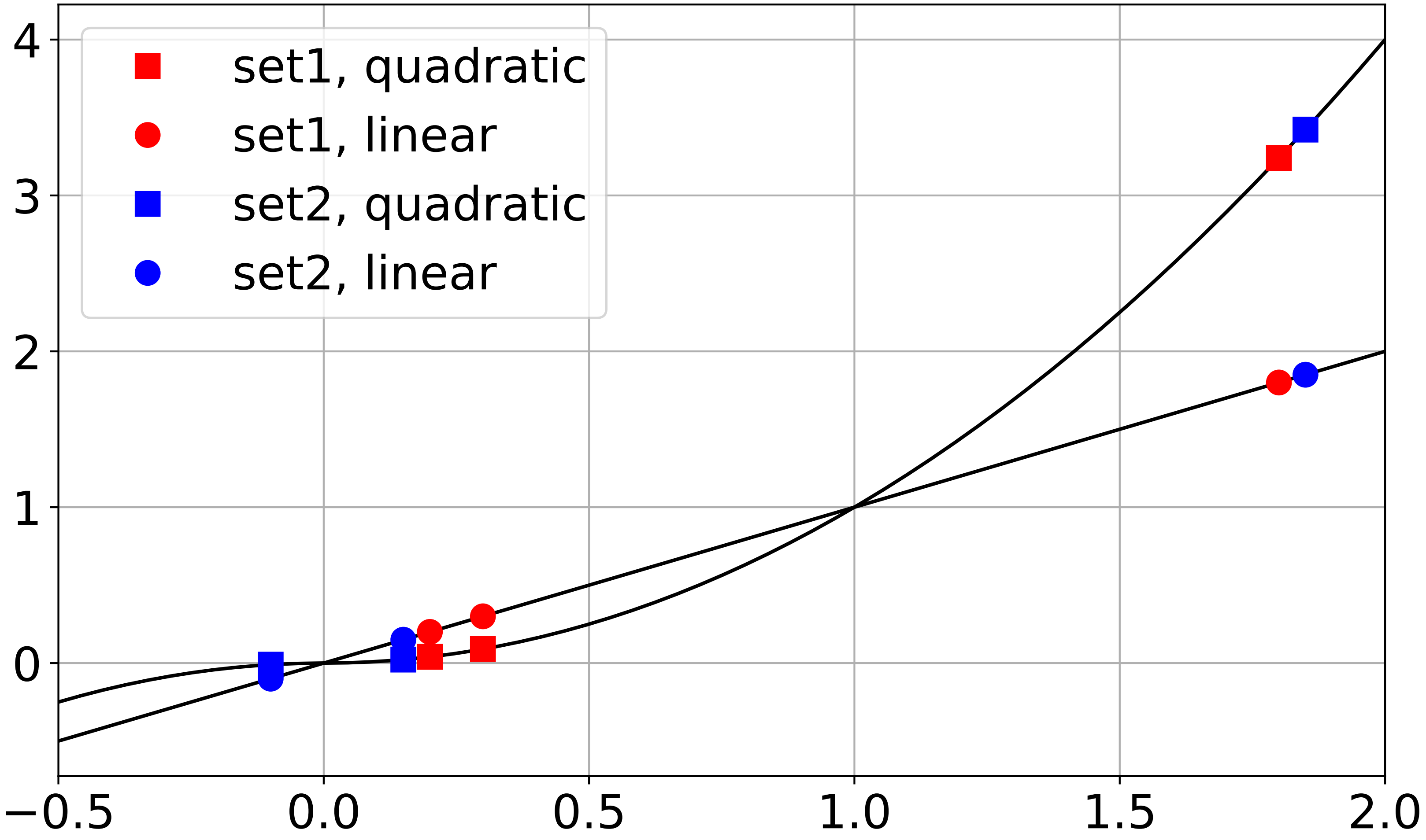

set1, quadratic
set1, linear
set2, quadratic
set2, linear
4
3
2
1
0
−0.5
0.0
0.5
1.0
1.5
2.0
