# Supplementary material for: Lipidomic Profile Reconstruction of Therapeutic Membrane Targets Using Physics‐Based Optimization with Limited Activity Data
Source: Adv Sci (Weinh). 2026 Sep 3;13(62):e76899. doi: 10.1002/advs.76899 (PMC13542398; doi:10.1002/advs.76899)
Supplement: Supplementary file 2 [file ADVS-13-e76899-s001.zip › SI/runtimeConvergence.pdf]

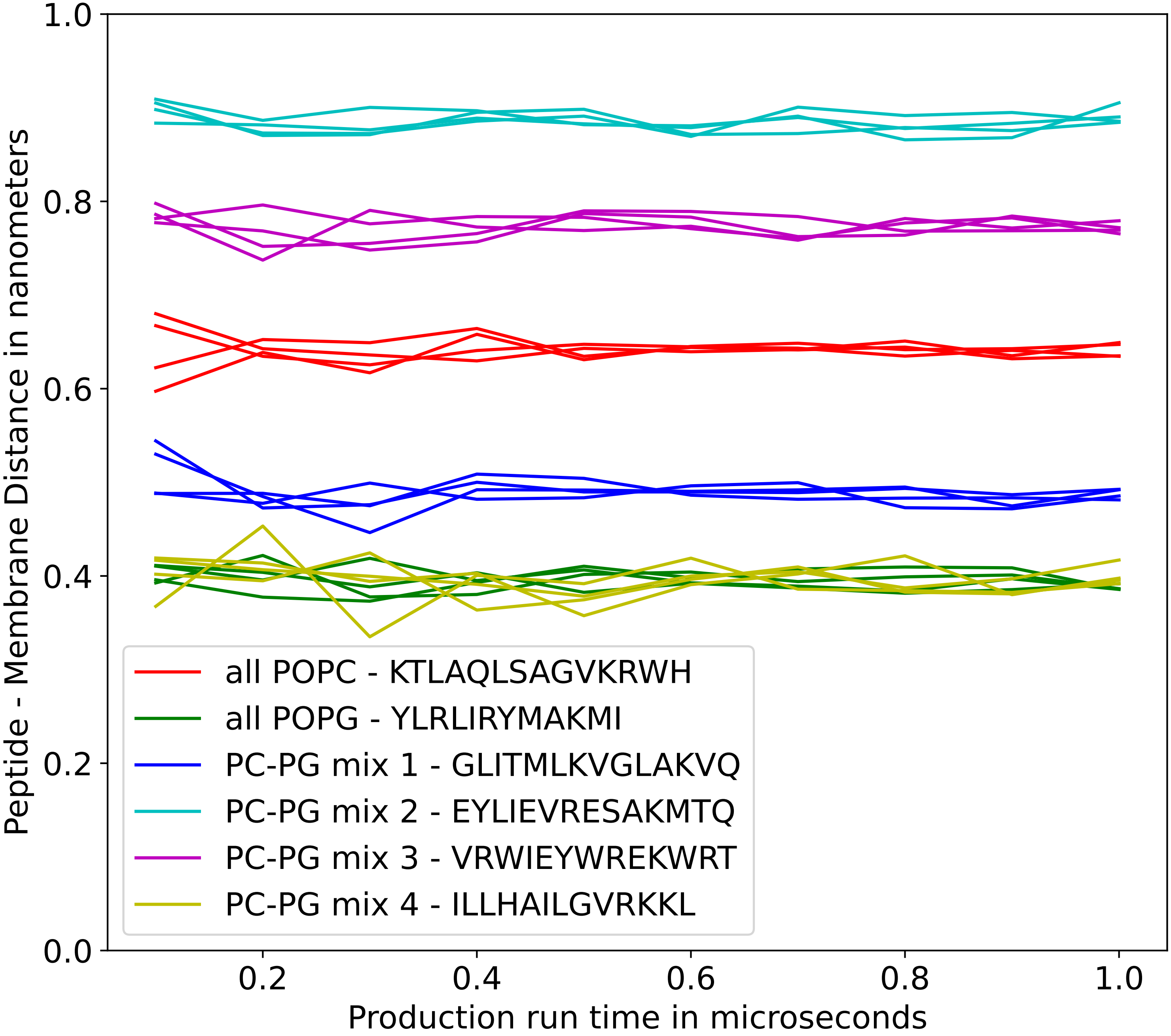

Peptide - Membrane Distance in nanometers
Production run time in microseconds
0.0
0.2
0.4
0.6
0.8
1.0
0.2
0.4
0.6
0.8
1.0
all POPC - KTLAQLSAGVKRWH
all POPG - YLRLIRYMAKMI
PC-PG mix 1 - GLITMLKVGLAKVQ
PC-PG mix 2 - EYLIEVRESAKMTQ
PC-PG mix 3 - VRWIEYWREKWRT
PC-PG mix 4 - ILLHAILGVRKKL
