# Supplementary material for: Lipidomic Profile Reconstruction of Therapeutic Membrane Targets Using Physics‐Based Optimization with Limited Activity Data
Source: Adv Sci (Weinh). 2026 Sep 3:e76899. Online ahead of print. doi: 10.1002/advs.76899 (PMC13542398; doi:10.1002/advs.76899)
Supplement: Supplementary file 1 [file ADVS-9999-e76899-s002.pdf]

Supplementary Information: Lipidomic Profile Reconstruction of Therapeutic Membrane Targets Using Physics-Based Optimization with Limited Activity Data

*Maximilian Krebs Herre Jelger Risselada**

Maximilian Krebs, Prof. Dr. Herre Jelger Risselada,
Address:
Condensed Matter Theory at the Physics Department
Technische Universität Dortmund
Otto-Hahn-Straße 4
Dortmund, Germany
Email Address: jelger.risselada@tu-dortmund.de

1 The role of the host values

As the following equation shows, the influence of the host values on the used fitness can be reduced to a constant shift.

$$\begin{aligned} F &= \sum_i w_i (x_{i,\text{host}} - x_{i,\text{target}}) \\ &= \underbrace{\sum_i w_i x_{i,\text{host}}}_{\text{const.}} - \sum_i w_i x_{i,\text{target}} \\ &= -\sum_i w_i x_{i,\text{target}} + \text{const.} \end{aligned}$$

Therefore, host values do not influence the ranking of different targets. This means that they do not affect the outcome of the optimization process. That behavior can be changed by introducing a monotonically increasing, nonlinear function f to adapt the fitness as follows:

$$F = \sum_i w_i f(x_{i,\text{host}} - x_{i,\text{target}})$$

This can be perceived as beneficial because it incorporates the non-toxic behavior of peptides directly into the optimization process. However, implementation has proven to be non-trivial. Using power law scaling, such as

$$f(x) = \text{sign(x)}|x|^\gamma$$

for $\gamma > 1$ causes Evo-MD to focus on specific peptides and neglect others during the membrane optimization process. For $0 < \gamma < 1$, on the other hand, Evo-MD has not yet demonstrated an increase in average fitness. ReLu-like functions might offer a promising alternative. However, if x starts in the negatives, this will remove the directionality from Evo-MD. For only positive values of x, at least for the classical ReLu, linear behavior is restored.

Additionally, using the non-linear scaling for the insertion depth by monotonically increasing functions only conserves the ranking of the individual insertion depths. This does not mean that the ranking of a summed fitness, as it is used for the optimization done in this work, is conserved as well. Some counter examples are shown in Figure S1. However, since the host membrane values still provide a valuable context for interpretation, we have decided to leave them in.

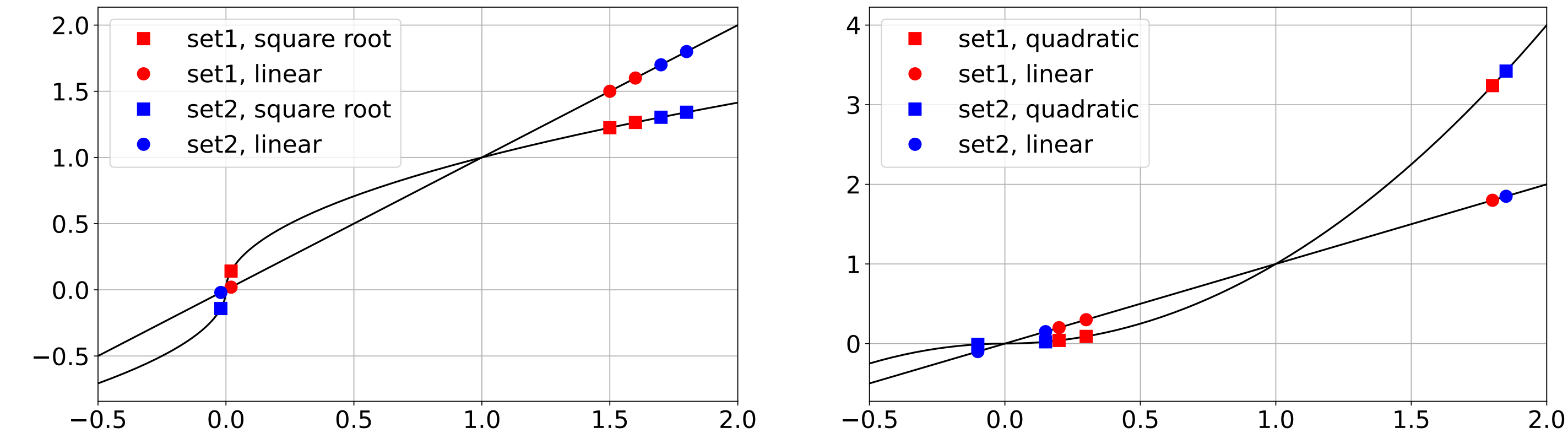

(a) Behaviour of fitness values for square root scaling. The red and the blue data points resemble two potential measurements of insertion depths, which are scaled and summed up for calculating the fitness. With linear scaling, the summed fitness of the blue set (3.48) is higher then the one of the red set (3.12). In the case of $\gamma = \frac{1}{2}$ scaling, the small increase of the value around $x = 0$ outweighs the decrease of both other values, leading to a higher fitness of the red set (2.631) over the blue set (2.504).

(b) Behaviour of fitness values for square quadratic scaling. The red and the blue data points resemble two potential measurements of insertion depths, which are scaled and summed up for calculating the fitness. With linear scaling, the summed fitness of the red set (2.3) is higher then the one of the blue set (1.9). In the case of $\gamma = 2$ scaling, the small increase of the value for high x outweighs the decrease of both other values, leading to a higher fitness of the blue set (3.435) over the red set (3.370).

Figure S1: **Examples of different scaling of the insertion depth changing the ranking of the summed fitness values.** It can be shown that even with (strictly) monotonically increasing scaling, while the order of the single insertion depth values remains conserved, the ranking by summed fitness is not.

2 Lipid Types

Given the anticipated prevalence of phosphatidylglycerol (PG), phosphatidylcholine (PC), and phosphatidylethanolamine (PE) as the most abundant lipids, the present study incorporates four conformations for each of them with the fatty acid tail groups: 1,2-dipalmitoyl (DP), 1,2-dioleoyl (DO), 1-palmitoyl-2-oleoyl (PO), and 1,2-dilauroyl (DL). Furthermore, one conformation of each of the following lipids is taken: sphingomyelin (DPSM), phosphatidylserine (DOPS) and ceramide (DPCE). Together with cholesterol (CHOL), this results in an alphabet of 16 different lipid types.

3 Convergence

To demonstrate the convergence of the algorithm, we show the development of reruns of the top-performing sequences from one run, compared to the slope of fitness development throughout one run. As shown in Figure S2, the variation of the fitness value of a single sequence is significantly higher than the increase in fitness after sampling 5000 sequences. Therefore, we don't expect a significant gain in a reasonable amount of time. In that sense, we consider this run to be converged.

4 Verification Data

The following plots, Figure S3 to S7, show the composition of the top 5 resulting membranes The following tables, Table S1 to S12, show the relative insertion depth Δx as well as the peptide-membrane distance x and the peptide-host-membrane distance x_{Host} for all training and verification peptides for the top 5 resulting membrane compositions as well as the standard 3:1 POPC:POPG model membrane. All values are averaged over three independent simulations. The optimal cutoff for the relative insertion depth was chosen as the midpoint between neighboring peptide values that offered the most correct classifications. Upright digits mark right classification, italic digits mark wrong classification. The cutoff for activity in absolute terms, has been empirically chosen as 0.7nm. Values that indicate activity are marked by a $*$, values that indicate toxicity are marked by a †.

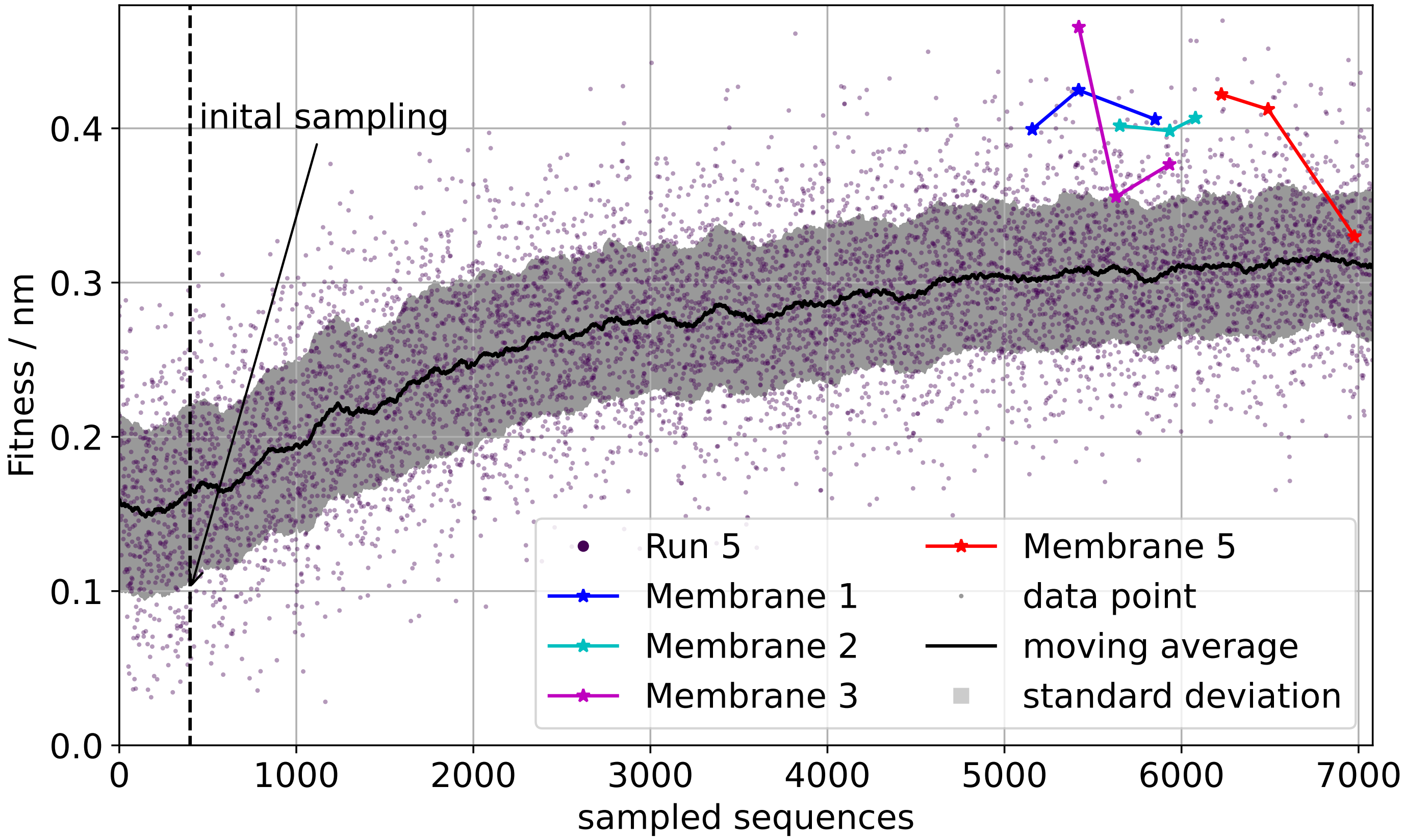

Figure S2: Convergence of fitness of ”run 5” is shown. This run produced the best individuals overall, including Membranes 1, 2, 3, and 5. Their occurrences in the Evo-MD history of Run 5 are highlighted. Particularly, the fitness variation of membrane 3 and membrane 5 reruns is high compared to the average fitness development at that time. There is also no gain in fitness from membrane 1 to any of the other membranes, despite the fact, that membrane 1 is the first ranked membrane to occur.

AMP	$\frac{\Delta x}{\text{nm}}$	$\frac{x}{\text{nm}}$	$\frac{x_{\text{Host}}}{\text{nm}}$	non AMP	$\frac{\Delta x}{\text{nm}}$	$\frac{x}{\text{nm}}$	$\frac{x_{\text{Host}}}{\text{nm}}$
GIHDILKYGKPS	*-0.005*	0.914	0.909	EYLIEVRESAKMTQ	-0.073	1.080	1.007
GLFDVIKKVASVIGGL	0.023	0.672*	0.695†	HILRMRIRQGMMT	-0.008	0.860	0.853
FCTMIPIPRCY	0.029	0.781	0.810	HILRMRIRQMMT	-0.002	0.835	0.833
LLPIVGNLLKSLL	0.032	0.656*	0.689†	HIRLMRIRQMMT	0.001	0.938	0.939
FLPLILRKIVTAL	0.045	0.662*	0.707	YQLLRIMRINIA	0.010	0.794	0.804
FLPLIGRVLSGIL	0.047	0.625*	0.672†	GLITMLKVGLAKVQ	0.011	0.753	0.764
FVQWFSKFLGRIL	0.051	0.659*	0.710	LRPAFKVSK	0.012	0.753	0.765
FLLFPLMCKIQGKC	0.055	0.687*	0.743	HIRAMRIRAQMMT	0.012	0.940	0.952
GLLGPLLKIAAKVGSNLL	0.069	0.558*	0.627†	KTLAQLSAGVKRWH	0.015	0.855	0.869
FFPIGVFCKIFKTC	0.108	0.622*	0.730	LIQVAPLGRLLKRR	*0.040*	0.795	0.834

Table S1: Verification for 25a75i. Optimal cutoff is 0.019.

AMP	$\frac{\Delta x}{\text{nm}}$	$\frac{x}{\text{nm}}$	$\frac{x_{\text{Host}}}{\text{nm}}$	non AMP	$\frac{\Delta x}{\text{nm}}$	$\frac{x}{\text{nm}}$	$\frac{x_{\text{Host}}}{\text{nm}}$
ILLHAILGVRKKL	*0.009*	0.742	0.752	HRALMRIRQCMT	0.009	0.849	0.858
FPLTWLKWWKWKK	0.029	0.909	0.939	YRAAMLRRQYMMT	0.020	0.950	0.969
YQLRLIMKYAI	0.047	0.715	0.762	HRAIMLRIRQMMT	0.022	0.881	0.904
YLRLIRYMAKMI	0.067	0.756	0.823	VRWIEYWREKWRT	0.023	0.919	0.941

Table S2: Rerun of the training data for 25a75i. Optimal cutoff is 0.026.

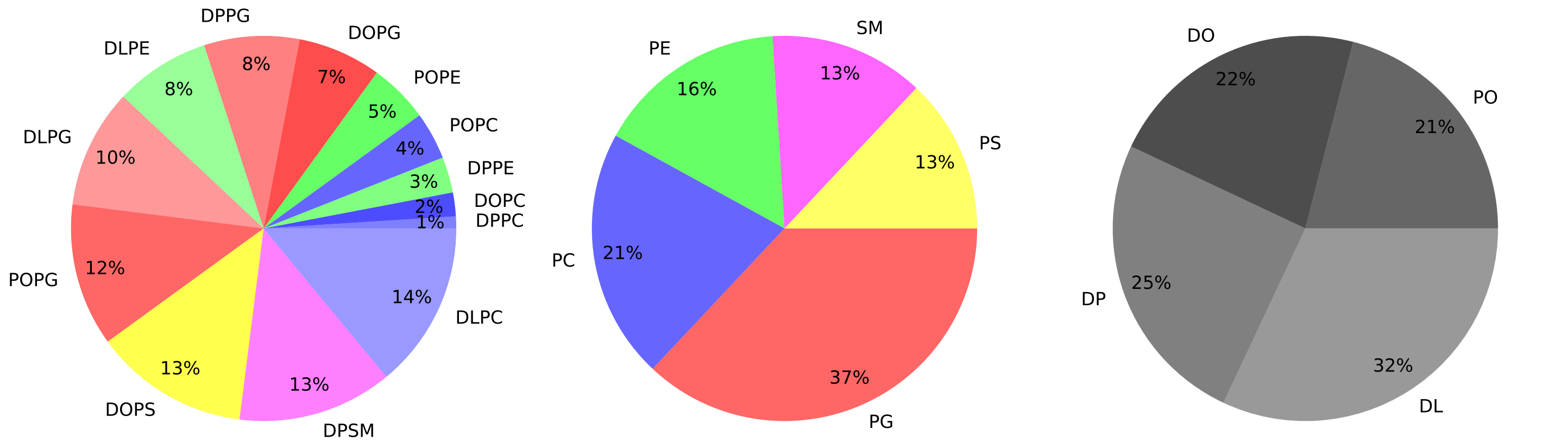

b) full composition a) composition by head groups c) composition by lipid tails

Figure S3: Membrane 1 Composition.

AMP	$\frac{\Delta x}{\text{nm}}$	$\frac{x}{\text{nm}}$	$\frac{x_{\text{Host}}}{\text{nm}}$	non AMP	$\frac{\Delta x}{\text{nm}}$	$\frac{x}{\text{nm}}$	$\frac{x_{\text{Host}}}{\text{nm}}$
GIHDILKYGKPS	*0.036*	0.874	0.909	EYLIEVRESAKMTQ	-0.048	1.055	1.007
FCTMIPIPRCY	*0.039*	0.771	0.810	HILRMRIRQGMMT	0.029	0.824	0.853
GLFDVIKKVASVIGGL	0.078	0.617*	$0.695^{\dagger}$	HIRAMRIRAQMMT	0.031	0.922	0.952
LLPIVGNLLKSLL	0.088	0.600*	$0.689^{\dagger}$	HIRLMRIRQMMT	0.049	0.890	0.939
FLLFPLMCKIQGKC	0.108	0.635*	0.743	LRPAFKVSK	0.054	0.711	0.765
FLPLILRKIVTAL	0.114	0.593*	0.707	HILRMRIRQMMT	0.054	0.778	0.833
FLPLIGRVLSGIL	0.114	0.558*	$0.672^{\dagger}$	KTLAQLSAGVKRWH	0.054	0.815	0.869
FVQWFSKFLGRIL	0.128	0.582*	0.710	YQLLRIMRINIA	0.063	0.742	0.804
GLLGPLLKIAAKVGSNLL	0.139	0.488*	$0.627^{\dagger}$	GLITMLKVGLAKVQ	0.067	0.697*	0.764
FFPIGVFCKIFKTC	0.173	0.558*	0.730	LIQVAPLGRLLKRR	*0.112*	0.723	0.834

Table S3: Verification for Membrane 1. Optimal cutoff is 0.072.

AMP	$\frac{\Delta x}{\text{nm}}$	$\frac{x}{\text{nm}}$	$\frac{x_{\text{Host}}}{\text{nm}}$	non AMP	$\frac{\Delta x}{\text{nm}}$	$\frac{x}{\text{nm}}$	$\frac{x_{\text{Host}}}{\text{nm}}$
FPLTWLKWWKWKK	0.075	0.864	0.939	YRAAMLRRQYMMT	0.030	0.940	0.969
ILLHAILGVRKKL	0.080	0.672*	0.752	VRWIEYWREKWRT	0.053	0.888	0.941
YQLRLIMKYAI	0.103	0.658*	0.762	HRALMRIRQCMT	0.060	0.798	0.858
YLRLIRYMAKMI	0.129	0.694*	0.823	HRAIMLRIRQMMT	0.070	0.833	0.904

Table S4: Rerun of the training data for Membrane 1. Optimal cutoff is 0.073.

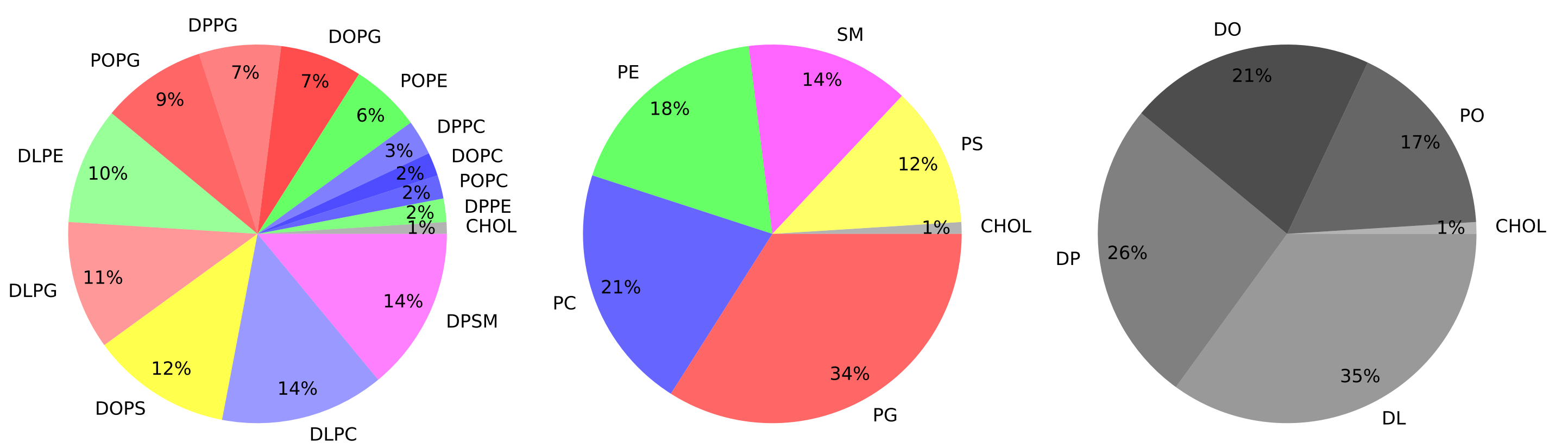

b) full composition a) composition by head groups c) composition by lipid tails

Figure S4: Membrane 2 Composition.

AMP	$\frac{\Delta x}{\text{nm}}$	$\frac{x}{\text{nm}}$	$\frac{x_{\text{Host}}}{\text{nm}}$	non AMP	$\frac{\Delta x}{\text{nm}}$	$\frac{x}{\text{nm}}$	$\frac{x_{\text{Host}}}{\text{nm}}$
GIHDILKYGKPS	*0.041*	0.868	0.909	EYLIEVRESAKMTQ	-0.036	1.043	1.007
FCTMIPIPRCY	*0.072*	0.738	0.810	HILRMRIRQGMMT	0.033	0.819	0.853
GLFDVIKKVASVIGGL	0.090	0.605*	$0.695^{\dagger}$	HIRLMRIRQMMT	0.052	0.887	0.939
LLPIVGNLLKSLL	0.098	0.590*	$0.689^{\dagger}$	LRPAFKVSK	0.058	0.707	0.765
FLPLILRKIVTAL	0.115	0.591*	0.707	HILRMRIRQMMT	0.059	0.773	0.833
FVQWFSKFLGRIL	0.127	0.583*	0.710	HIRAMRIRAQMMT	0.062	0.891	0.952
FLLFPLMCKIQGKC	0.129	0.614*	0.743	YQLLRIMRINIA	0.062	0.742	0.804
FLPLIGRVLSGIL	0.131	0.541*	$0.672^{\dagger}$	GLITMLKVGLAKVQ	0.070	0.694*	0.764
GLLGPLLKIAAKVGSNLL	0.145	0.482*	$0.627^{\dagger}$	KTLAQLSAGVKRWH	0.074	0.795	0.869
FFPIGVFCKIFKTC	0.185	0.545*	0.730	LIQVAPLGRLLKRR	*0.099*	0.735	0.834

Table S5: Verification for Membrane 2. Optimal cutoff is 0.082.

AMP	$\frac{\Delta x}{\text{nm}}$	$\frac{x}{\text{nm}}$	$\frac{x_{\text{Host}}}{\text{nm}}$	non AMP	$\frac{\Delta x}{\text{nm}}$	$\frac{x}{\text{nm}}$	$\frac{x_{\text{Host}}}{\text{nm}}$
FPLTWLKWWKWKK	0.081	0.858	0.939	YRAAMLRRQYMMT	0.063	0.907	0.969
ILLHAILGVRKKL	0.084	0.667*	0.752	VRWIEYWREKWRT	0.064	0.877	0.941
YQLRLIMKYAI	0.116	0.646*	0.762	HRALMRIRQCMT	0.074	0.784	0.858
YLRLIRYMAKMI	0.138	0.685*	0.823	HRAIMLRIRQMMT	0.079	0.825	0.904

Table S6: Verification for Membrane 2. Optimal cutoff is 0.080.

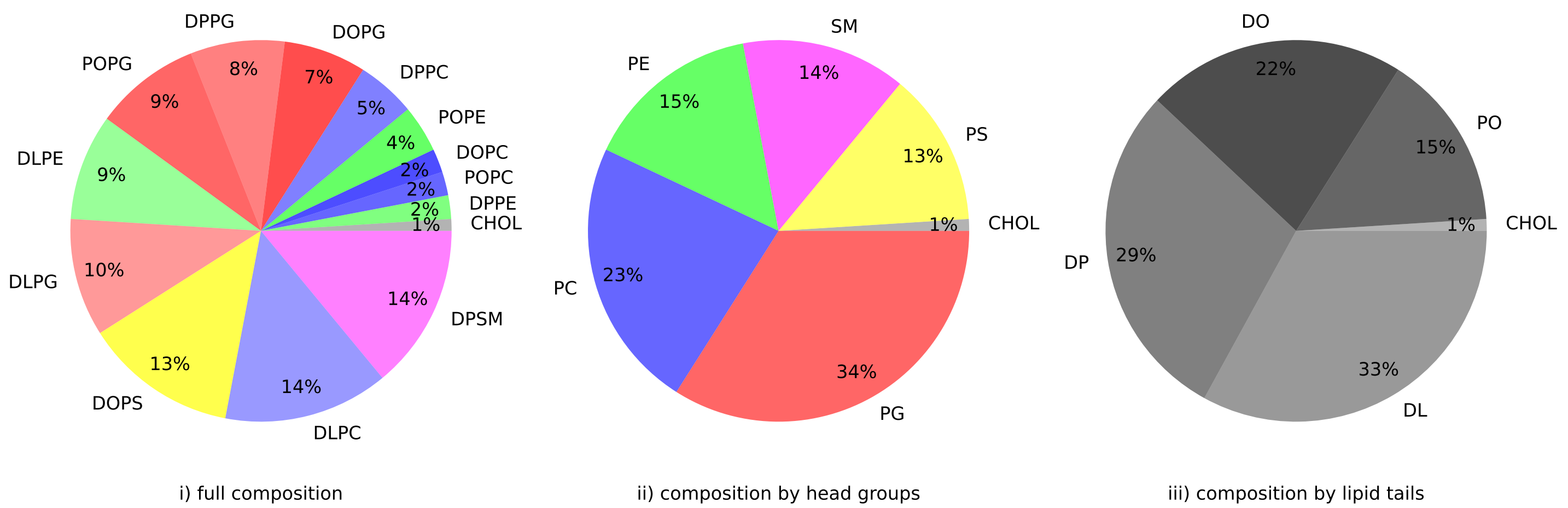

Figure S5: Membrane 3 Composition.

AMP	$\frac{\Delta x}{\text{nm}}$	$\frac{x}{\text{nm}}$	$\frac{x_{\text{Host}}}{\text{nm}}$	non AMP	$\frac{\Delta x}{\text{nm}}$	$\frac{x}{\text{nm}}$	$\frac{x_{\text{Host}}}{\text{nm}}$
GIHDILKYGKPS	*0.032*	0.878	0.909	EYLIEVRESAKMTQ	-0.043	1.050	1.007
FCTMIPIPRCY	*0.064*	0.746	0.810	HILRMRIRQGMMT	0.034	0.818	0.853
GLFDVIKKVASVIGGL	0.088	0.607*	$0.695^{\dagger}$	LRPAFKVSK	0.051	0.714	0.765
LLPIVGNLLKSLL	0.094	0.594*	$0.689^{\dagger}$	HILRMRIRQMMT	0.052	0.780	0.833
FLPLILRKIVTAL	0.108	0.599*	0.707	HIRLMRIRQMMT	0.054	0.885	0.939
FLLFPLMCKIQGKC	0.108	0.635*	0.743	GLITMLKVGLAKVQ	0.063	0.701	0.764
FVQWFSKFLGRIL	0.120	0.590*	0.710	KTLAQLSAGVKRWH	0.063	0.806	0.869
FLPLIGRVLSGIL	0.124	0.549*	$0.672^{\dagger}$	YQLLRIMRINIA	0.065	0.739	0.804
GLLGPLLKIAAKVGSNLL	0.147	0.480*	$0.627^{\dagger}$	HIRAMRIRAQMMT	0.067	0.885	0.952
FFPIGVFCKIFKTC	0.179	0.551*	0.730	LIQVAPLGRLLKRR	*0.103*	0.731	0.834

Table S7: Verification for Membrane 3. Optimal cutoff is 0.07765.

AMP	$\frac{\Delta x}{\text{nm}}$	$\frac{x}{\text{nm}}$	$\frac{x_{\text{Host}}}{\text{nm}}$	non AMP	$\frac{\Delta x}{\text{nm}}$	$\frac{x}{\text{nm}}$	$\frac{x_{\text{Host}}}{\text{nm}}$
ILLHAILGVRKKL	*0.069*	0.683*	0.752	YRAAMLRRQYMMT	0.054	0.915	0.969
FPLTWLKWWKWKK	0.076	0.862	0.939	VRWIEYWREKWRT	0.062	0.879	0.941
YQLRLIMKYAI	0.111	0.651*	0.762	HRALMRIRQCMT	0.067	0.791	0.858
YLRLIRYMAKMI	0.126	0.696*	0.823	HRAIMLRIRQMMT	0.074	0.830	0.904

Table S8: Rerun of training data for Membrane 3. Optimal cutoff is 0.075.

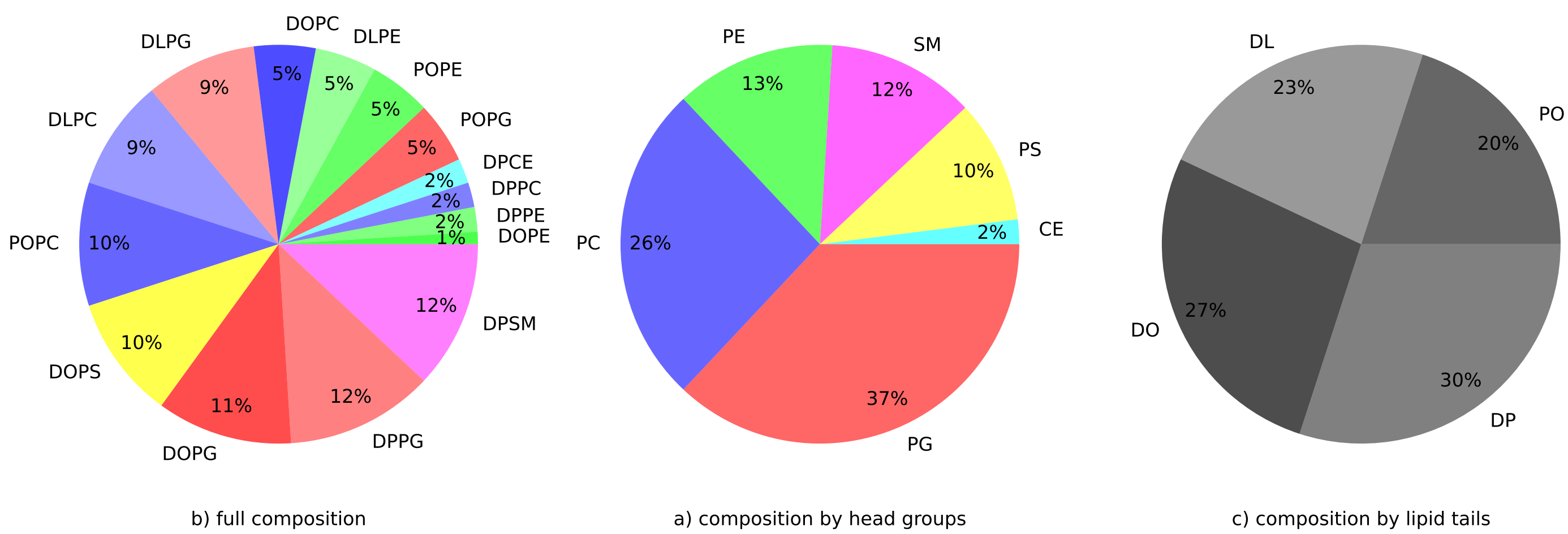

Figure S6: Membrane 4 Composition.

AMP	$\frac{\Delta x}{\text{nm}}$	$\frac{x}{\text{nm}}$	$\frac{x_{\text{Host}}}{\text{nm}}$	non AMP	$\frac{\Delta x}{\text{nm}}$	$\frac{x}{\text{nm}}$	$\frac{x_{\text{Host}}}{\text{nm}}$
FCTMIPIPRCY	*0.043*	0.768	0.810	EYLIEVRESAKMTQ	-0.037	1.044	1.007
GIHDILKYGKPS	*0.045*	0.865	0.909	HILRMRIRQGMMT	0.033	0.819	0.853
GLFDVIKKVASVIGGL	0.080	0.615*	$0.695^{\dagger}$	HIRLMRIRQMMT	0.048	0.891	0.939
LLPIVGNLLKSLL	0.094	0.595*	$0.689^{\dagger}$	LRPAFKVSK	0.050	0.715	0.765
FLPLILRKIVTAL	0.101	0.605*	0.707	HIRAMRIRAQMMT	0.053	0.899	0.952
FLLFPLMCKIQGKC	0.112	0.631*	0.743	GLITMLKVGLAKVQ	0.060	0.704	0.764
FLPLIGRVLSGIL	0.123	0.549*	$0.672^{\dagger}$	KTLAQLSAGVKRWH	0.065	0.805	0.869
FVQWFSKFLGRIL	0.125	0.585*	0.710	HILRMRIRQMMT	0.065	0.768	0.833
GLLGPLLKIAAKVGSNLL	0.144	0.483*	$0.627^{\dagger}$	YQLLRIMRINIA	0.067	0.737	0.804
FFPIGVFCKIFKTC	0.164	0.567*	0.730	LIQVAPLGRLLKRR	0.079	0.756	0.834

Table S9: Verification for Membrane 4. Optimal cutoff is 0.079.

AMP	$\frac{\Delta x}{\text{nm}}$	$\frac{x}{\text{nm}}$	$\frac{x_{\text{Host}}}{\text{nm}}$	non AMP	$\frac{\Delta x}{\text{nm}}$	$\frac{x}{\text{nm}}$	$\frac{x_{\text{Host}}}{\text{nm}}$
FPLTWLKWWKWKK	0.074	0.865	0.939	YRAAMLRRQYMMT	0.047	0.922	0.969
ILLHAILGVRKKL	0.076	0.675*	0.752	VRWIEYWREKWRT	0.057	0.885	0.941
YQLRLIMKYAI	0.090	0.671*	0.762	HRALMRIRQCMT	0.060	0.798	0.858
YLRLIRYMAKMI	0.119	0.703	0.823	HRAIMLRIRQMMT	0.069	0.835	0.904

Table S10: Rerun of training data for Membrane 4. Optimal cutoff is 0.071.

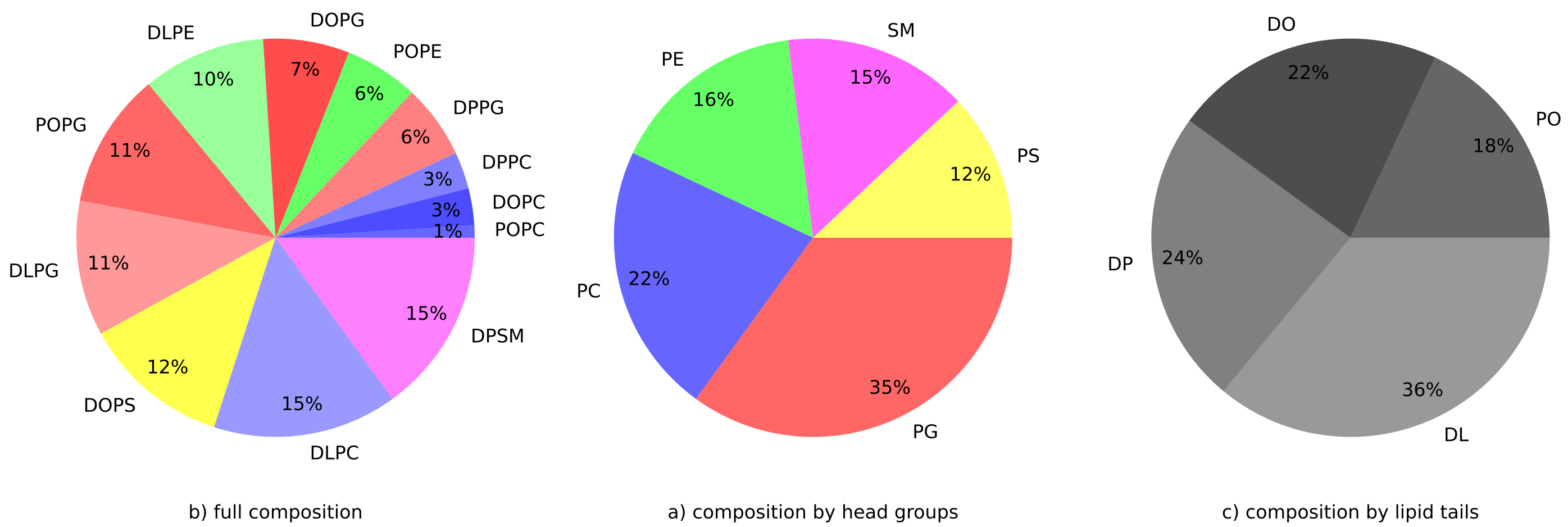

Figure S7: Membrane 5 Composition.

AMP	$\frac{\Delta x}{\text{nm}}$	$\frac{x}{\text{nm}}$	$\frac{x_{\text{Host}}}{\text{nm}}$	non AMP	$\frac{\Delta x}{\text{nm}}$	$\frac{x}{\text{nm}}$	$\frac{x_{\text{Host}}}{\text{nm}}$
GIHDILKYGKPS	*0.023*	0.887	0.909	EYLIEVRESAKMTQ	-0.047	1.055	1.007
FCTMIPIPRCY	*0.026*	0.784	0.810	HILRMRIRQGMMT	0.004	0.848	0.853
GLFDVIKKVASVIGGL	*0.060*	0.635*	$0.695^{\dagger}$	LRPAFKVSK	0.024	0.741	0.765
LLPIVGNLLKSLL	0.069	0.620*	$0.689^{\dagger}$	HIRAMRIRAQMMT	0.031	0.921	0.952
FLLFPLMCKIQGKC	0.081	0.662*	0.743	HILRMRIRQMMT	0.035	0.798	0.833
FLPLIGRVLSGIL	0.094	0.578*	$0.672^{\dagger}$	HIRLMRIRQMMT	0.038	0.901	0.939
FLPLILRKIVTAL	0.095	0.612*	0.707	GLITMLKVGLAKVQ	0.043	0.721	0.764
FVQWFSKFLGRIL	0.102	0.608*	0.710	YQLLRIMRINIA	0.045	0.759	0.804
GLLGPLLKIAAKVGSNLL	0.106	0.521*	$0.627^{\dagger}$	KTLAQLSAGVKRWH	0.048	0.821	0.869
FFPIGVFCKIFKTC	0.142	0.588*	0.730	LIQVAPLGRLLKRR	0.060	0.774	0.834

Table S11: Verification for Membrane 5. Optimal cutoff is 0.064.

AMP	$\frac{\Delta x}{\text{nm}}$	$\frac{x}{\text{nm}}$	$\frac{x_{\text{Host}}}{\text{nm}}$	non AMP	$\frac{\Delta x}{\text{nm}}$	$\frac{x}{\text{nm}}$	$\frac{x_{\text{Host}}}{\text{nm}}$
ILLHAILGVRKKL	0.051	0.700	0.752	YRAAMLRRQYMMT	0.023	0.946	0.969
FPLTWLKWWKWKK	0.058	0.880	0.939	HRALMRIRQCMT	0.037	0.821	0.858
YQLRLIMKYAI	0.090	0.671*	0.762	VRWIEYWREKWRT	0.040	0.901	0.941
YLRLIRYMAKMI	0.107	0.716	0.823	HRAIMLRIRQMMT	0.048	0.856	0.904

Table S12: Rerun of training data for Membrane 5. Optimal cutoff is 0.049.

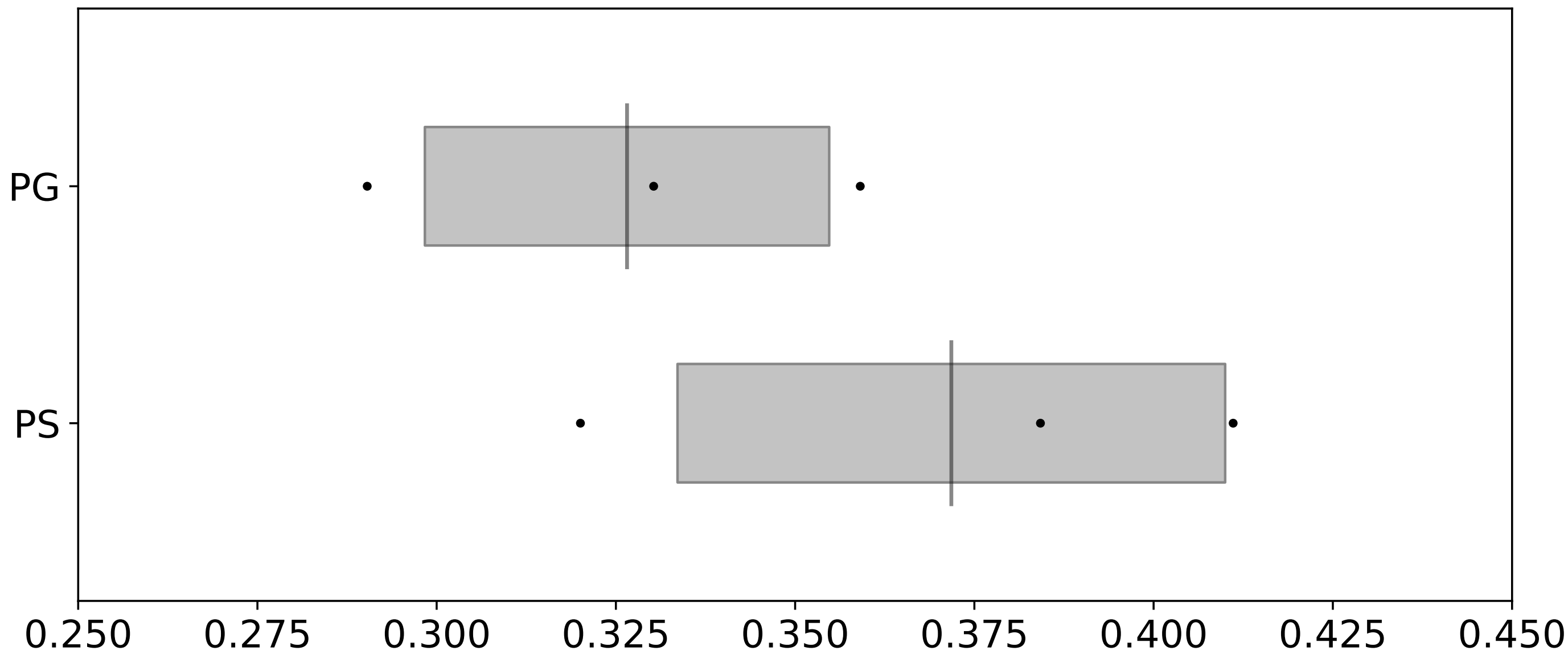

Figure S8: Fitness comparison between the negatively charged lipids PS and PG. The black dots are the fitness values for Membrane 3 with all PS head groups replaced by PG, referred to as PG, and Membrane 3 with all PG head groups replaced by PS, referred to as PS. The grey box marks the standard deviation, the grey line within it the mean value of the fitness.

5 PG and PS lipids

To illustrate the difference between the PG and PS lipid headgroups, we replaced all the PS lipids in Membrane 3 (Figure S5) with PG lipids, and vice versa. We then calculated the fitness of these two membranes multiple times and compared them, as shown in Figure S8. The small difference between the two membranes indicates that the PS headgroup may be slightly preferred. However, the difference between these membranes is not significant. Therefore, we conclude that our model cannot properly resolve the difference between these headgroups at the coarse-grained level.

6 Convergence of the peptide membrane distance

While the exact distance between peptide and membrane varies over time, the average distance should converge over time. By approximating the positions of membrane and peptide as Gaussians (Figure 2 main text), we can express this convergence via the central limit theorem. To explore the sampling time needed, for the Gaussians to converge, we took a look at a few example systems S9, which have been sampled for different times.

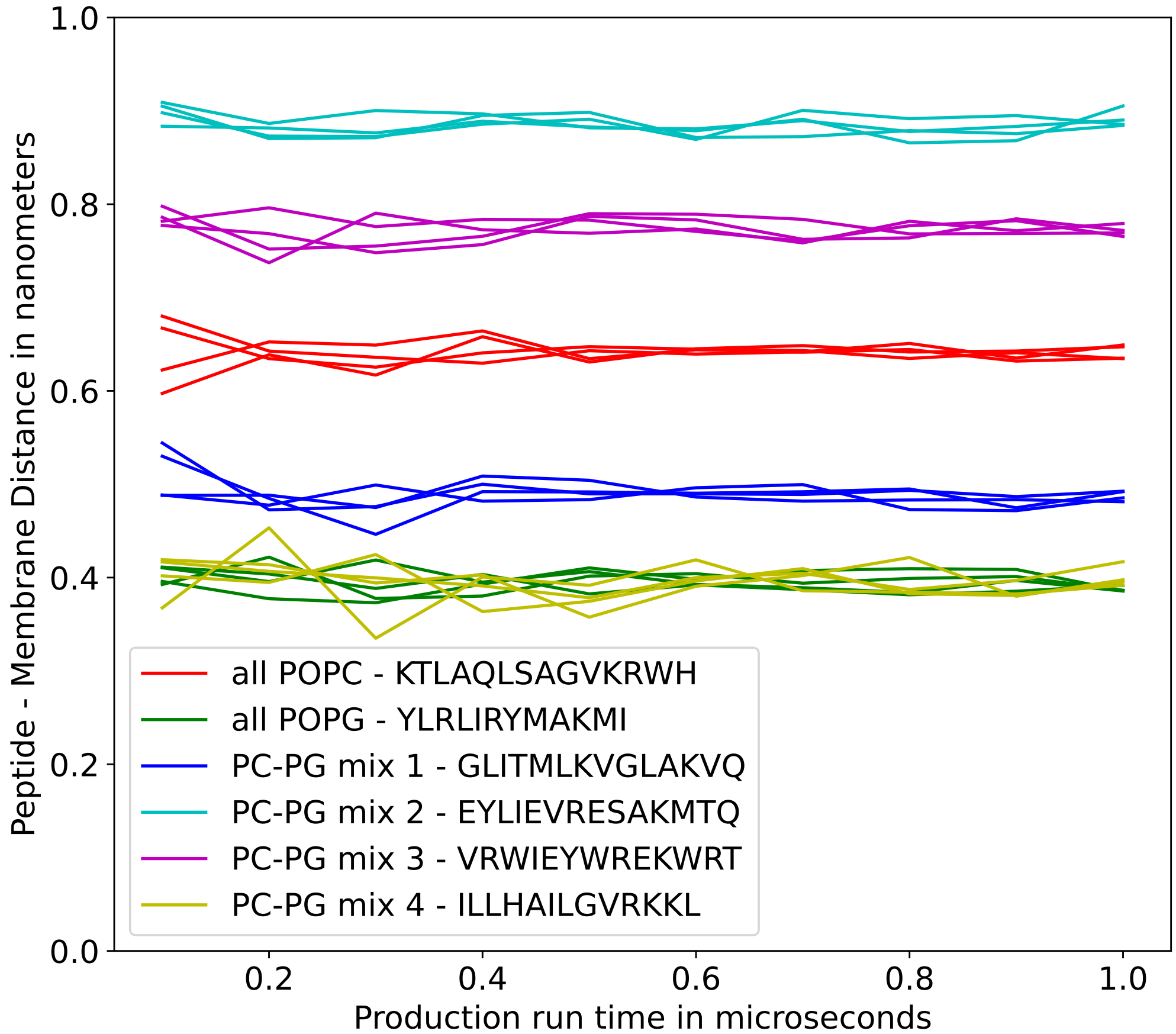

Figure S9: Runtime convergence check of the peptide membrane distance for reruns of different peptide membrane systems. Each system was run four times and the sampling times were evaluated between 100 ns and 1 µs in 100 nsincrements. The insertion depth appears to be converged at roughly 600 ns for all the systems. To account for potential outliers, we sampled for 900 ns in all simulations performed in this study.
